# Genetic polymorphism in *ATG16L1* gene is associated with adalimumab use in inflammatory bowel disease

**DOI:** 10.1186/s12967-017-1355-9

**Published:** 2017-12-11

**Authors:** V. J. A. A. Nuij, M. P. Peppelenbosch, C. J. van der Woude, G. M. Fuhler

**Affiliations:** Department of Gastroenterology and Hepatology, Erasmus MC, University Medical Center Rotterdam, ‘s-Gravendijkwal 230, 3015 CE Rotterdam, The Netherlands

**Keywords:** Inflammatory bowel disease, Crohn’s disease, Ulcerative colitis, Genetics, *ATG16L1*, Anti-TNFα

## Abstract

**Background:**

The role of single nucleotide polymorphisms (SNPs) associated with inflammatory bowel disease (IBD) is gaining interest. With the advent of novel therapies, personalized treatment in IBD is a future goal. We wondered whether IBD-associated SNPs are able to predict response to anti-TNFα treatment.

**Methods:**

Data on treatment use and primary response, loss of response and side effects to anti-TNFα treatments were retrieved for 570 IBD patients. rs13361189 (*IRGM*), rs10210302 (*ATG16L1*), rs2066844, rs2066845, rs2066847 (*NOD2*), rs35873774 (XBP1), rs11175593 (*LRRK2*), rs11465804 (*IL23R*), rs2301436 (*CCR6*), rs744166 (*STAT3*) and rs4821544 (*NCF4*) SNP status were determined.

**Results:**

No associations were found between genetic variants of the *LRRK2*, *CCR6*, *IL23R* and *NCF4* genes and response to anti-TNFα. For *NOD2* and *XBP1* associations were found, however, these associations were not strong enough to survive multiple testing corrections. Strikingly, patients carrying the *ATG16L1* T300A variant were more likely to be treated with adalimumab, even after correction for disease phenotype, disease behavior and age (p = 0.004, OR 2.8, CI 1.6–5.0).

**Conclusions:**

Genetic polymorphisms in the known IBD-associated gene *ATG16L1* correlate with requirement of treatment, suggesting a different IBD disease phenotype in these patients. Further investigation will need to elucidate the implications of these findings and identify the underlying disease characteristics.

**Electronic supplementary material:**

The online version of this article (10.1186/s12967-017-1355-9) contains supplementary material, which is available to authorized users.

## Background

Inflammatory Bowel diseases, comprising of Crohn’s disease (CD), Ulcerative colitis (UC) and IBD-unclassified (IBD-U), are multifactorial in their etiology.

In the last two decades, the introduction of the anti-tumor necrosis factor-α (anti-TNFα) drugs (e.g. infliximab and adalimumab) has expanded the treatment arsenal with drugs potent for both inducing remission as well as maintaining remission [1–3]. Although these immunosuppressants are increasingly used, the need for intestinal surgery remains unchanged, with 25–61% of newly diagnosed IBD requiring surgery at least once within the first 5 years after diagnosis [4–6]. Additionally, anti-TNFα treatments are not effective in 20–40% of IBD patients and loss of response and/or is seen in a substantial proportion of the patients [1, 7–10]. While it has been suggested that earlier treatment with anti-TNFα could improve disease outcome [11], we were previously unable to confirm a benefit for earlier anti-TNFα treatment on IBD-disease complications [12]. The most likely explanation appeared to be that an inappropriate selection of patients eligible for therapy had led to suboptimal treatment and subsequently outcome.

Genetics play an important role in IBD, as IBD shows a large hereditary component and genetic variants may influence cellular functions, the innate immune system and thereby both disease activity and response to treatment [13, 14]. Genome-wide association studies (GWAS) have identified over 163 single-nucleotide polymorphisms (SNPs) that are associated with IBD [15, 16]. Based on their known function in normal cellular settings, efforts have been made to cluster these SNPs into functional categories in order to glean insight into the mechanistic aspects of IBD pathology [17, 18]. Key pathways identified so far are innate immunity, defective epithelial barrier, autophagy, IL10 signaling and adaptive immunity, although these definitions are not always clear-cut, with many genes acting in more than one of these categories. While the general role of these genes in cellular processes is in most cases known, it is as yet largely unclear how the IBD-associated SNPs in these genes affect cellular function, or how such changed cellular functions would contribute to the development of IBD. However, there are some positive exceptions. For instance, the IBD-associated variants in the *NOD2*, *ATG16L1* and *IRGM* genes affect cellular autophagy processes and bacterial clearance in (innate) immune cells, and may affect bacterial composition of the gut in patients with IBD [19–22]. In addition, SNPs in the interleukin 23 receptor gene (*IL23R*) were recently shown to affect expression of the anti-microbial peptide DMBT1 in intestinal epithelial cells in IBD [23]. Our own studies demonstrated that an IBD-associated SNP in the neutrophil cytosolic factor 4 (*NCF4*) gene results in a decreased antimicrobial function of granulocytes, as demonstrated by a reduced production of reactive oxygen species by these cells [24]. In light of the immune-cell modulatory properties of several of the known IBD-associated SNPs, it is likely that these SNPs may affect response of patients to immune-modulatory drugs as well.

Identifying associations between patients’ genetics and characteristics and response to treatment would open up the possibility of implementing personalised treatment strategies. Tailored strategies in the future could include treatment according to the individual patients’ genetic profile. With this study we aimed to identify patients likely to benefit from anti-TNFα treatment, based on their genetic profile.

## Methods

All patients of whom DNA was available at the Erasmus MC University Medical Center, and the diagnosis of IBD could be confirmed according to the Lennard-Jones criteria, were included in this study [25]. Disease characteristics were scored according to the Montreal criteria [26]. Patients having had a liver transplantation or suffering from auto-immune hepatitis or PSC and who were treated for these conditions, were excluded from analysis. Data on patient and disease characteristics were obtained from the patients’ medical charts. For each patient, the following characteristics were retrieved: date of birth, date of IBD diagnosis, age at diagnosis, date of first visit to the Erasmus MC University Medical Centre, IBD phenotype, gender, comorbidities, familial IBD status, disease location, disease behavior, extra-intestinal manifestations, fistula and abscesses and the amount of surgery. SNP carriers were compared to non-SNP carriers for above mentioned parameters. For disease location and behavior, patients with IBDU were classified as patients with UC. Maximum disease extension and disease characterization were scored according to the Montreal criteria [26]. Number of flares during follow-up were scored based on clinical and endoscopic parameters. End of follow-up was January 1st, 2013. This study was approved by the institutional medical ethical board of the Erasmus MC (MEC-2012-245).

Of all patients, records pertaining to infliximab (IFX) and adalimumab (ADA) use were perused. Of the treatments, their use, side-effects and primary non-response and loss of response were evaluated. Primary nonresponse to biologic therapy was defined as an absence of symptomatic improvement with persistently high levels of C-reactive protein (CRP) after induction treatment. Long-term sustained response to biologics was defined as improvement of the symptoms lasting at least 1 year without any further adjustments of the therapeutic regimen. Failure of the therapeutic regimen was defined by an absence of improvement of the symptoms of disease and by a decision of the treating physician to add steroids, add another immunosuppressor, switch to another immunosuppressor, switch to another anti-TNF medication (adalimumab), or refer for CD-related surgery.

Several immune-regulatory genes were selected for this study. We focused on genes involved in innate immunity/autophagy/bacterial clearance in both in blood immune cells and Paneth cells (*IRGM*, *NOD2*, *LRRK2*, *ATG16L1*, *XBP1* and *NCF4*) [19, 24, 27, 28] and genes affecting adaptive immune responses (*IL23R*, *STAT3*, *CCR6*) [29, 30]. Rs13361189 (*IRGM*), rs10210302 (*ATG16L1*), rs2066844, rs2066845, rs2066847 (*NOD2*), rs35873774 (XBBP1), rs11175593 (*LRRK2*), rs11465804 (*IL23R*), rs2301436 (*CCR6*), rs744166 (*STAT3*) and rs4821544 (*NCF4*) SNP status were determined by KBiosciences, UK. Inconclusive SNP analyses were excluded, accounting for the variable number of patients analyzed per SNP (Additional file 1: Table S1). Different SNPs in *NOD2* were combined for analysis, as these SNPS have been shown to alter cellular function in a similar manner [31, 32]. Due to the low number of patients homozygous for the SNPs, patients heterozygous for the risk allele were analysed together with patients carrying two risk alleles of the same gene, thereby comparing carriers and non-carriers of the IBD-associated alleles.

Statistical analyses were performed using descriptive statistics, independent t tests, Mann–Whitney nonparametric tests, Chi square (Χ^2^) tests and Fisher’s exact test. Independent samples t tests were used to compare means. Proportions were compared using the Χ^2^ test or Fisher’s exact test. Two-sided p values < 0.05 were considered significant. Associations were assessed using a logistic regression using the enter method expressed as odds ratios (OR) with 95% confidence interval (CI). Correction for multiple testing was applied to logistic regression analysis, with two-sided p values of < 0.0055 considered significant correction for multiple testing. Overall logistic analysis associating IFX to the SNPs in the IBD related genes were corrected for age, IBD subtype and fistulising disease. Subanalyses in CD patients were corrected for fistula and age. Subanalyses for UC were corrected for age. Logistic analyses aiming to associate ADA to the SNPs were corrected for extra-intestinal manifestations, age, and IBD subtype. Subanalyses in CD patients were corrected for extra-intestinal manifestations, fistula and age. Subanalyses in UC patients were corrected for extra-intestinal manifestations and age. Statistical analyses were performed using SPSS for Windows software (v23.0, Chicago, IL).

## Results

### Patient and disease characteristics

Of the 591 eligible patients, 19 were excluded due to liver disease or liver transplantation, one patient was not suffering from IBD, and one patient was excluded because of multiple kidney transplantations, leaving a total of 570 patients. Thede included 411 CD (71.9%), 148 UC (26.0%) and 11 IBDU (1.9%) patients. Patient and disease characteristics are shown in Table 1. The median age at IBD diagnosis was 27 years (range 5–79). Median age at diagnosis was 25 years in CD, 26 years in UC, and 32 years in IBDU. Of our patients 54.7% were female. The mean duration of follow-up was 9.2 years (range 0.1–49.1). Four patients developed colorectal cancer during follow-up.

**Table 1:** Patient characteristics

	Total N = 570	CD N = 411	UC N = 148	IBDU N = 11
Follow-up				
Length, mean, range	9.2 yrs (range 0.1–49.1)	9.5 yrs (range 0.1–49.1)	8.6 yrs (range 0.2–32.3)	4.9 yrs (range 0.8–10.5)
Length, median	6.9 yrs	7.0 yrs	6.9 yrs	4.8 yrs
Gender				
Male	258 (45.3)	169 (41.1)	85 (57.4)	4 (36.4)
Female	312 (54.7)	242 (58.9)	63 (42.6)	7 (63.6)
Age at diagnosis				
Mean, range	27 yr (range 5–79)	27 yr (range 5–79)	28 yr (range 8–69)	32 yr (range 17–47)
Median	25 yr	25 yr	26 yr	32 yr
Disease location CD				
L1—terminal ileum		99 (24.1)		
L2—colon		90 (21.9)		
L3—ileocolon		120 (29.2)		
(+) L4—upper GI tract		63 (15.3)		
Other		0 (0)		
Unknown		21 (5.1)		
No inflammation		18 (4.4)		
Disease location UC				
E1—proctitis	8 (5.0)		7 (4.7)	1 (9.1)
E2—left sided colitis	69 (43.4)		64 (43.2)	5 (45.4)
E3—pancolitis	73 (45.9)		69 (46.6)	4 (36.4)
No inflammation	3 (1.9)		3 (2.0)	0 (0)
Other	2 (1.3)		1 (0.7)	1 (9.1)
Unknown	4 (2.5)		4 (2.7)	0
Backwash ileitis	35		22	3
Rectal sparing	19		16	1
Disease behaviour CD				
B1—luminal disease		126 (30.7)		
B2—stenosis		89 (21.7)		
B3—abscesses and/or fistula		181 (44.0)		
P—perianal disease		144 (35.0)		
Unknown		15 (3.6)		
Extra-intestinal	172 (30.2)	134 (32.6)	36 (24.3)	2 (18.2)
Family history of IBD				
Yes	127 (22.3)	98 (23.8)	25 (16.9)	4 (36.4)
No	380 (66.7)	267 (65.0)	108 (73.0)	5 (45.4)
Not documented	63 (11.0)	46 (11.2)	15 (10.1)	2 (18.2)
Family history of CRC				
Yes	36 (6.3)	22 (5.4)	13 (8.8)	1 (9.1)
No	469 (82.3)	342 (83.2)	119 (80.4)	8 (72.7)
Not documented	65 (11.4)	47 (11.4)	16 (10.8)	2 (18.2)

All numbers are presented as n (%), unless stated otherwise

*CD* Crohn’s disease, *UC* ulcerative colitis, *IBDU* unclassified inflammatory bowel disease, *CRC* colorectal cancer, *IBD* inflammatory bowel disease, *yrs* years

In total 211 patients were treated with IFX and 179 with ADA, with 111 patients receiving both treatments. A total of 126 patients developed side effects while treated with IFX and 89 did so while on ADA. Fifty-nine patients experienced loss of response to IFX and 26 to ADA. Twenty-seven patients were primary non-responders on IFX and 14 never responded to ADA.

CD patients were more likely to be treated with IFX (p = 0.022, OR 1.6, CI 1.1–2.3) or ADA (p < 0.0001, OR 7.6, CI 4.2–13.9) compared to UC/IBDU patients. IFX treated patients were less likely to achieve mucosal healing than patients not requiring this medication (p < 0.0001, OR 0.48). Both IFX (p < 0.0001, OR 2.5) and ADA (p = 0.002, OR 1.8) treated patients were more likely to undergo bowel resection, compared to patients not receiving these medications. However these associations were no longer significant after correction for fistula and disease phenotype. Furthermore ADA treated patients suffered from extra-intestinal manifestations (p < 0.012) more often than patients who were not treated with ADA, which remained significant after correction for disease phenotype (p = 0.047, OR 1.5, CI 1.0–2.2).

### Genetics

SNPs in nine IBD related genes were evaluated. Distribution of the genetic profiles can be found in Table 2. The minor allele frequency known in literature and the frequency in this cohort can be found in Table 3.

**Table 2:** Genetic profile patients

Gene	SNP	Total	Homozygous: no SNP	Heterozygous, one SNP	Homozygous, two SNPs
*IRGM*	C	568	430	120	18
*ATG16L1*	T	559	103	272	184
*NOD2*	T, C, C	570	428	128	14
rs2066844	T	559	484	71	4
rs2066845	C	567	539	27	1
rs2066847	C	561	513	48	0
*XBP1*	C, protective	567	510	54	3
*LRRK2*/*MUC19*	T	562	532	30	0
*CCR6*	A	558	135	280	143
*IL23R*	G, protective	564	524	38	2
*STAT3*	T	562	218	266	78
*NCF4*	C	570	282	225	63

Number of patients that could be analysed for each gene, and the distribution of risk alleles in these genes. All numbers are expressed as n

**Table 3:** Minor allele frequencies of investigated SNPs in general population (controls), as reported for IBD (IBD) and in the IBD cohort described here (cohort)

Treatment	SNP	MAF controls [31]	MAF IBD [31]	MAF cohort
*IRGM*	C	0.13	0.18	0.14
*ATG16L1*	T	0.48	0.40 [44]	0.57
*NOD2*	T, C, C	–		–
rs2066844	T	0.07	0.14	0.07
rs2066845	C	0.01	0.05	0.03
rs2066847	C	0.02	0.11	0.04
*XBP1*	C, protective	0.04	0.04	0.05
*LRRK2*/*MUC19*	T	0.02	0.03	0.03
*CCR6*	A	0.47	0.48	0.50
*IL23R*	G, protective	0.08	0.02	0.04
*STAT3*	T	0.42	0.41	0.38
*NCF4*	C	0.33	0.38 [31, 45, 46]	0.31

*MAF* minor allele frequency, *SNP* single nucleotide polymorphism

An association between the *ATG16L1* risk allele and Crohn’s disease (p = 0.007) and younger age at IBD diagnosis (26.83 vs 29.93, p = 0.032) was observed. Sub analysis per disease phenotype showed that UC or IBDU patients carrying the *ATG16L1* risk allele were less likely to achieve mucosal healing (p = 0.027). In CD patients, *ATG16L1* SNP carriers were significantly more likely to have a family member with IBD (p = 0.004).

Carrying the *IRGM* risk allele was associated with male gender (p = 0.034) and younger age at diagnosis (25.84 vs 27.90, p = 0.048). Sub analysis for IBD phenotype did not how any differences between SNP carriers and non-carriers.

*NOD2* risk allele carriers more often suffered from CD (p = 0.002) and carrying the *NOD2* SNP was associated with colonic disease in these patients (p < 0.0001).

Carrying the *CCR6* SNP was associated with achievement of mucosal healing in UC patients (p = 0.009).

CD patients carrying the *NCF4* SNP were more likely to suffer from stenosing disease (p = 0.005, OR 2.0, CI 1.2–3.2), even after correction for age (p = 0.004, OR 2.0, CI 1.2–3.3), although no association with fistula or non-stenosis-non-fistulising disease was observed.

*IL23R* CD SNP patients were also more likely to suffer from stenosing disease (p = 0.023).

There were no differences in basic clinical parameters between patients carrying risk alleles of the *XBP1*, *STAT3* and *LRRK2*/MUC2 genes and patients who did not carry these risk alleles.

None of the risk alleles were associated with the number of flared (corrected for follow-up time).

### Genetics versus treatments

The only significant association between anti-TNFα use and genetic risk variants was found for *ATG16L1*. While there were no associations between *ATG16L1* risk allele carriers and IFX use/response, neither in the entire cohort, nor in CD and UC sub analyses, IBD patients carrying the *ATG16L1* SNP were significantly more prone to use ADA (p = 0.004, OR 2.4, CI 1.3–4.4, corrected for age, extra-intestinal manifestations, IBD subtype and multiple testing). In CD patients, this correlation also remained true (p = 0.005 OR 2.6 CI 1.3–5.0).

Other, nominally significant associations are listed below.

Logistic regression analysis after correction for age and IBD phenotype showed that patients carrying the *IRGM* risk allele were more prone to develop primary non response to IFX (OR 2.4, CI 1.0–5.7, p = 0.048). In sub analysis per disease phenotype, it was apparent that in particular UC patients carrying the SNP in the *IRGM* gene were more prone to suffer from primary non response to IFX (p = 0.009, OR 12.2, CI 1.2–78.8, corrected for age) whereas there were no associations between the *IRGM* risk allele and IFX use and response in CD patients (corrected for age and fistula).

Regarding ADA, patients carrying the *IRGM* risk allele more often used ADA, compared to patients who did not carry this risk allele (p = 0.021, OR 0.58, CI 0.36–0.92 corrected for age, IBD subtype and extra intestinal manifestations). Sub analysis for CD patients only did not show any associations between the *IRGM* SNP and ADA.

*XBP1* risk allele carriers responded less to IFX (p = 0.016, OR 3.7, CI 1.2–10.8) and patients carrying a *STAT3* SNP more often had side effects on IFX (p = 0.021, OR 0.30, CI 0.11–0.83). No associations were found between the risk allele carriers of the *NOD2*, *LRRK2*/*MUC19*, *CCR6*, *IL23R* and *NCF4* genes and response to anti-TNFα. As only 13 UC + IBDU patients were treated with ADA, no further analysis could be performed. An overview of the outcomes of the logistic regression can be found in Table 4.

**Table 4:** SNP versus treatment logistic regression

Treatment	*IRGM*	*ATG16L1*	*NOD2*	*XBP1*	LRRK2/MUC19	CCR6	IL23	*STAT3*	NCF4
Requirement									
Infliximab	ns	ns	OR 0.62, CI 0.41–0.95	ns	ns	ns	ns	ns	ns
Adalimumab	OR 0.57, CI 0.36–0.91	OR 2.4, CI 1.3–4.4	ns	ns	ns	ns	ns	ns	ns
Side-effects									
Infliximab	ns	ns	ns	ns	ns	ns	ns	OR 0.19, CI 0.05–0.78	ns
Adalimumab	ns	ns	ns	ns	ns	ns	ns	ns	ns
Loss of response									
Infliximab	ns	ns	ns	ns	ns	ns	ns	ns	ns
Adalimumab	ns	ns	ns	ns	ns	ns	ns	ns	ns
Primary non response									
Infliximab	OR 2.8, CI 1.1–7.0	ns	ns	OR 3.8, CI 1.2–12.0	ns	ns	ns	ns	ns
Adalimumab	ns	ns	ns	ns	ns	ns	ns	ns	ns

Logistic regression analysis showing the association between the different IBD risk genes and clinical response to anti-TNFα treatments

*SNP* single nucleotide polymorphism, *ns* not significant, *OR* odds ratio, *CI* confidence interval

## Discussion

Personalized medicine for IBD is called for, as a lack of identification of the appropriate patient group may result in underestimation of clinical results of some treatments. For instance, studies with the granulocyte colony stimulating factor (GM-CSF) sargramostim have been controversial [33–35]. However, granulocytes of CD patients carrying the *NCF4* risk allele were recently shown to be less sensitive to stimulation with GM-CSF, suggesting that only a subpopulation of patients may actually benefit from this treatment [24]. Similarly, trials on the use of interferon-β-1α (IFN-β-1α) in IBD initially did not seem to be effective [36, 37], while on closer inspection, Croatian and Russian patients were shown to have high remission and response rates using this treatment, while Western European populations experienced the opposite [36]. These studies emphasise the diversity in IBD, and suggest that in study populations, genetic variants could be used to stratify groups of patients, potentially leading to a tailored treatment model.

The purpose of our study was to investigate putative links between IBD-risk alleles and the effect of anti-TNFα treatment, something which has received relatively little attention to date. For five of the nine investigated genes, no association was found with treatment response, whereas with three other genes (*IRGM*, *XBP1*, *STAT3*) associations were found, but were not strong enough to survive multiple testing corrections. The strongest association observed was a tendency for *ATG16L1* SNP carrying patients to be treated with ADA, with increased odds for using ADA when carrying two *ATG16L1* risk alleles (not shown). Recently, a retrospective study of 588 IBD patients investigated 41 IBD risk genes, including the nine in the current study, and showed that only the *XBP1* variant was nominally associated with start of IFX/ADA [38]. However, unlike the current study, this study was performed in a pediatric cohort. Interestingly, a prospective study testing 31 risk-alleles (including *NOD2*, *ATG16L1* and *IL23R*) in 102 patients showed that only polymorphisms in *ATG16L1* correlated to clinical response, with patients carrying the *ATG16L1* risk alleles having significantly better response to ADA. Although *ATG16L1* SNP status was associated with use of ADA in our study, we did not find primary non-response, side-effects or loss or response to anti-TNFα to be modulated by any of the SNPs studied. However, unlike the other three measured outcomes, treatment use in itself is not likely a parameter that is influenced by patient genetics, as treatment of patients is decided by physicians and SNP status in this study was not available to treating physicians. Hence, these results hint at an underlying disease phenotype which is not captured by the currently used parameters (Montreal score, disease location, disease severity). If true, this would suggest that based on genetics, it might be possible to define patient groups with subtly different disease, that cannot otherwise be distinguished, which opens up an interesting avenue of investigation. Attempts have already been made to categorize CD patients into genetic-based Crohn’s disease subgroups according to SNPs in 46 disease susceptibility loci [39]. Surprisingly, these genetic-based subgroups could not be explained by clinical phenotypic variables such as disease location and behaviour, suggesting that patients had been categorized into previously un-identified sub-groups by genetically-determined pathways rather than the currently used classifications that are mostly based on disease location [39].

A direct comparison between genetic studies is complicated by differences in either cohort size, disease phenotype and age of onset. Furthermore, differences in genetic associations between disease location and behaviour might be found in a different minor allele frequency distribution between cohorts. While *NOD2* and *ATG16L1* variants are some of the most consistently observed IBD-associated loci, association studies differ per cohort. We have investigated only nine of the ~ 200 IBD related risk genes, while interactions between SNPs in different genes might also account for specific phenotypes [40–42]. Only around 13 and 8.2% of disease variance of CD and UC, respectively, has been explained by the risk loci identified to date, and other genes or epigenetic events may contribute to various extents in these cohorts [15, 43].

## Conclusions

In conclusion, genetic polymorphisms in the *ATG16L1* gene correlate with ADA treatment, for which a previously unidentified disease phenotype may be responsible. This suggests that genetic make-up of IBD patients may in future help physicians decide on personalized treatment strategies. Further investigation will need to elucidate the implications of these findings and identify the corresponding disease phenotype.

## Additional file

**Additional file 1: Table S1.** SNPs versus treatment, number of patients per group.

## References

[1] Hanauer SB, Feagan BG, Lichtenstein GR (2002). Maintenance infliximab for Crohn’s disease: the ACCENT I randomised trial. Lancet.

[2] Sandborn WJ, Hanauer SB, Rutgeerts P (2007). Adalimumab for maintenance treatment of Crohn’s disease: results of the CLASSIC II trial. Gut.

[3] Rutgeerts P, Sandborn WJ, Feagan BG (2005). Infliximab for induction and maintenance therapy for ulcerative colitis. N Engl J Med.

[4] Loftus EV, Schoenfeld P, Sandborn WJ (2002). The epidemiology and natural history of Crohn’s disease in population-based patient cohorts from North America: a systematic review. Aliment Pharmacol Ther.

[5] Cosnes J, Nion-Larmurier I, Beaugerie L (2005). Impact of the increasing use of immunosuppressants in Crohn’s disease on the need for intestinal surgery. Gut.

[6] Sands BE, Arsenault JE, Rosen MJ (2003). Risk of early surgery for Crohn’s disease: implications for early treatment strategies. Am J Gastroenterol.

[7] Farrell RJ, Alsahli M, Jeen Y-T (2003). Intravenous hydrocortisone premedication reduces antibodies to infliximab in Crohn’s disease: a randomized controlled trial. Gastroenterology.

[8] Colombel J-F, Loftus EV, Tremaine WJ (2004). The safety profile of infliximab in patients with Crohn’s disease: the Mayo clinic experience in 500 patients. Gastroenterology.

[9] Hanauer SB, Sandborn WJ, Rutgeerts P (2006). Human anti-tumor necrosis factor monoclonal antibody (adalimumab) in Crohn’s disease: the CLASSIC-I trial. Gastroenterology.

[10] Ford AC, Sandborn WJ, Khan KJ (2011). Efficacy of biological therapies in inflammatory bowel disease: systematic review and meta-analysis. Am J Gastroenterol.

[11] Peyrin-Biroulet L, Bigard M-A, Malesci A, Danese S (2008). Step-up and top-down approaches to the treatment of Crohn’s disease: early may already be too late. Gastroenterology.

[12] Nuij V, Fuhler GM, Edel AJ (2015). Benefit of earlier anti-TNF treatment on IBD disease complications?. J Crohn’s Colitis.

[13] Van Limbergen J, Russell RK, Nimmo ER, Satsangi J (2007). The genetics of inflammatory bowel disease. Am J Gastroenterol.

[14] Orholm M, Fonager K, Sorensen HT (1999). Risk of ulcerative colitis and Crohn’s disease among offspring of patients with chronic inflammatory bowel disease. Am J Gastroenterol.

[15] Liu JZ, van Sommeren S, Huang H (2015). Association analyses identify 38 susceptibility loci for inflammatory bowel disease and highlight shared genetic risk across populations. Nat Genet.

[16] Jostins L, Ripke S, Weersma RK (2012). Host–microbe interactions have shaped the genetic architecture of inflammatory bowel disease. Nature.

[17] Ek WE, D’Amato M, Halfvarson J (2014). The history of genetics in inflammatory bowel disease. Ann Gastroenterol.

[18] Lees CW, Barrett JC, Parkes M, Satsangi J (2011). New IBD genetics: common pathways with other diseases. Gut.

[19] Deuring JJ, Fuhler GM, Konstantinov SR (2014). Genomic ATG16L1 risk allele-restricted Paneth cell ER stress in quiescent Crohn’s disease. Gut.

[20] Parkes M (2012). Evidence from genetics for a role of autophagy and innate immunity in IBD pathogenesis. Dig Dis.

[21] Fritz T, Niederreiter L, Adolph T (2011). Crohn’s disease: NOD2, autophagy and ER stress converge. Gut.

[22] Knights D, Silverberg MS, Weersma RK (2014). Complex host genetics influence the microbiome in inflammatory bowel disease. Genome Med.

[23] Diegelmann J, Czamara D, Le Bras E (2013). Intestinal DMBT1 expression is modulated by Crohn’s disease-associated IL23R variants and by a DMBT1 variant which influences binding of the transcription factors CREB1 and ATF-2. PLoS ONE.

[24] Somasundaram R, Deuring JJ, van der Woude CJ (2012). Linking risk conferring mutations in NCF4 to functional consequences in Crohn’s disease. Gut.

[25] Lennard-Jones JE (1989). Classification of inflammatory bowel disease. Scand J Gastroenterol.

[26] Satsangi J, Silverberg MS, Vermeire S, Colombel J-F (2006). The Montreal classification of inflammatory bowel disease: controversies, consensus, and implications. Gut.

[27] Kaser A, Lee A-H, Franke A (2008). XBP1 links ER stress to intestinal inflammation and confers genetic risk for human inflammatory bowel disease. Cell.

[28] Tschurtschenthaler M, Adolph TE, Ashcroft JW (2017). Defective ATG16L1-mediated removal of IRE1α drives Crohn’s disease-like ileitis. J Exp Med.

[29] Pidasheva S, Trifari S, Phillips A (2011). Functional studies on the IBD susceptibility gene IL23R implicate reduced receptor function in the protective genetic variant R381Q. PLoS ONE.

[30] Ito T, Carson WF, Cavassani KA (2011). CCR6 as a mediator of immunity in the lung and gut. Exp Cell Res.

[31] Jung C, Colombel J-F, Lemann M (2012). Genotype/phenotype analyses for 53 Crohn’s disease associated genetic polymorphisms. PLoS ONE.

[32] Barrett JC, Hansoul S, Nicolae DL (2008). Genome-wide association defines more than 30 distinct susceptibility loci for Crohn’s disease. Nat Genet.

[33] Takazoe M, Matsui T, Motoya S (2009). Sargramostim in patients with Crohn’s disease: results of a phase 1–2 study. J Gastroenterol.

[34] Korzenik JR, Dieckgraefe BK (2005). An open-labelled study of granulocyte colony-stimulating factor in the treatment of active Crohn’s disease. Aliment Pharmacol Ther.

[35] Korzenik JR, Dieckgraefe BK, Valentine JF (2005). Sargramostim for active Crohn’s disease. N Engl J Med.

[36] Pena-Rossi C, Schreiber S, Golubovic G (2008). Clinical trial: a multicentre, randomized, double-blind, placebo-controlled, dose-finding, phase II study of subcutaneous interferon-beta-la in moderately active ulcerative colitis. Aliment Pharmacol Ther.

[37] Rossi CP, Hanauer SB, Tomasevic R (2009). Interferon beta-1a for the maintenance of remission in patients with Crohn’s disease: results of a phase II dose-finding study. BMC Gastroenterol.

[38] Jakobsen C, Cleynen I, Andersen PS (2014). Genetic susceptibility and genotype–phenotype association in 588 Danish children with inflammatory bowel disease. J Crohns Colitis.

[39] Cleynen I, John JMM, Henckaerts L (2010). Molecular reclassification of Crohn’s disease by cluster analysis of genetic variants. PLoS ONE.

[40] Wagner J, Sim WH, Ellis JA (2010). Interaction of Crohn’s disease susceptibility genes in an Australian paediatric cohort. PLoS ONE.

[41] Plantinga TS, Crisan TO, Oosting M (2011). Crohn’s disease-associated ATG16L1 polymorphism modulates pro-inflammatory cytokine responses selectively upon activation of NOD2. Gut.

[42] Plantinga TS, Joosten LAB, Netea MG (2011). ATG16L1 polymorphisms are associated with NOD2-induced hyperinflammation. Autophagy.

[43] Ellinghaus D, Bethune J, Petersen B-S, Franke A (2015). The genetics of Crohn’s disease and ulcerative colitis—*status quo* and beyond. Scand J Gastroenterol.

[44] Wellcome Trust Case Control C (2007). Genome-wide association study of 14,000 cases of seven common diseases and 3,000 shared controls. Nature.

[45] Cleynen I, Vermeire S (2015). The genetic architecture of inflammatory bowel disease: past, present and future. Curr Opin Gastroenterol.

[46] Rioux JD, Xavier RJ, Taylor KD (2007). Genome-wide association study identifies new susceptibility loci for Crohn disease and implicates autophagy in disease pathogenesis. Nat Genet..

