# Supplementary material for: Genetic polymorphism in ATG16L1 gene is associated with adalimumab use in inflammatory bowel disease
Source: J Transl Med. 2017 Dec 11;15:248. doi: 10.1186/s12967-017-1355-9 (PMC5725822; doi:10.1186/s12967-017-1355-9)
Supplement: Supplementary file 1 — Additional file 1: Table S1. SNPs versus treatment, number of patients per group. [file 12967_2017_1355_MOESM1_ESM.docx]

**Table S1│ SNPs versus treatment, number of patients per group.**

| **Treatment** | ***IRGM*** | ***ATG16L1*** | ***NOD2*** | ***XBP1*** | ***LRRK2*/*MUC19*** | ***CCR6*** | ***IL23R*** | ***STAT3*** | ***NCF4*** |
| --- | --- | --- | --- | --- | --- | --- | --- | --- | --- |
| ***Total nr of patients*** | 568 | 559 | 570 | 567 | 562 | 558 | 564 | 562 | 570 |
| ***Requirement*** |  |  |  |  |  |  |  |  |  |
| **Infliximab** | 210 | 205 | 211 | 209 | 207 | 206 | 209 | 207 | 211 |
| **Adalimumab** | 178 | 172 | 179 | 177 | 176 | 172 | 175 | 174 | 179 |
| ***Side-effects*** |  |  |  |  |  |  |  |  |  |
| **Infliximab** | 125 | 120 | 126 | 125 | 125 | 123 | 124 | 122 | 126 |
| **Adalimumab** | 89 | 84 | 89 | 88 | 87 | 85 | 86 | 84 | 89 |
| ***Loss of response*** |  |  |  |  |  |  |  |  |  |
| **Infliximab** | 59 | 58 | 59 | 58 | 56 | 55 | 58 | 57 | 59 |
| **Adalimumab** | 25 | 24 | 26 | 25 | 26 | 25 | 25 | 26 | 26 |
| ***Primary non response*** |  |  |  |  |  |  |  |  |  |
| **Infliximab** | 27 | 26 | 27 | 27 | 27 | 27 | 27 | 27 | 27 |
| **Adalimumab** | 14 | 13 | 14 | 14 | 14 | 14 | 14 | 14 | 14 |

Total number of patients that could be analysed per SNP, and their distribution amongst specific treatments and reaction to treatment. All numbers are expressed as n. SNP: single nucleotide polymorphism.
